# Clinical analysis of surgery for type III esophageal atresia via thoracoscopy: a study of a Chinese single-center experience

**DOI:** 10.1186/s13019-020-01097-z

**Published:** 2020-03-30

**Authors:** Jianqin Zhang, Qiang Wu, Liu Chen, Yunjin Wang, Xu Cui, Wenhua Huang, Chaoming Zhou

**Affiliations:** 1 Department of Pediatric Surgery, Fujian Maternity and Child Health Hospital, Affiliated Hospital of Fujian Medical University, Fuzhou, 350001 P. R. China; 2 Department of Pediatric Surgery, The First Affiliated Hospital of Fujian Medical University, Fuzhou, 350001 P. R. China

**Keywords:** Thoracoscopic surgery, Traditional surgery, Type III esophageal

## Abstract

**Purpose:**

The purpose of this study was to investigate the effectiveness and safety of the operation for type III esophageal atresia using a thoracoscope.

**Methods:**

The clinical data for 92 patients with type III esophageal atresia in our hospital from January 2015 to December 2018 were analyzed retrospectively. There were 49 patients in group A who underwent thoracoscopic surgery and 43 patients in group B who underwent conventional surgery.

**Results:**

The mechanical ventilation time (55.7 ± 11.4 h vs 75.6 ± 19.2 h), intensive care time (3.6 ± 1.8d vs 4.7 ± 2.0d), postoperative hospitalization time (13.1 ± 2.2d vs 16.8 ± 4.3d), thoracic drainage volume (62.7 ± 25.5 ml vs 125.4 ± 46.1 ml), blood transfusion volume (30.5 ± 10.4 ml vs 55.3 ± 22.7 ml) and surgical incision length (2.0 ± 0.5 cm vs 8.0 ± 1.8 cm) in group A were lower than those in group B, and the differences were statistically significant (*P* < 0.05). Among the postoperative complications, the incidences of postoperative severe pneumonia (8.2% vs 23.3%), poor wound healing (2.0% vs 14.0%) and chest wall deformity (0% vs 11.6%) in group A were significantly lower than those in group B (*P* < 0.05). There was no significant difference in the incidence of anastomotic stricture, tracheomalacia or gastroesophageal reflux between the two groups after surgery and early during follow-up (*P* > 0.05), and there were no complications such as achalasia signs and esophageal diverticulum in either group.

**Conclusion:**

Surgery for type III esophageal atresia via thoracoscopy has the same safety and clinical effectiveness as traditional surgery and has the advantages of smaller incision and chest wall deformity.

## Introduction

Congenital esophageal atresia is one of the most common congenital malformations in newborns, with morbidities of 1:2500 to 4500, and type III esophageal atresia is the most common type, with an incidence of approximately 85% [1–4]. In 1941, the first operation for esophageal atresia was completed by Haight and Towsplet [5], and since then, conventional open surgery has been developed and widely recognized. The traditional operation is safe and effective, but it has some shortcomings, including substantial trauma, bleeding, slow postoperative recovery, postoperative thoracic deformity, and intercostal nerve injury [6, 7]. With the maturity and progress of surgical technology and the development of surgical instruments, as well as requirements among patients for an aesthetically pleasing surgical incision and early recovery after the operation, minimally invasive surgery is becoming increasingly popular in various fields. After more than 10 years of exploration and practice, thoracoscopic esophageal atresia has been widely carried out worldwide [6, 8–13]. In this study, the clinical data for patients with type III esophageal atresia in our hospital were analyzed retrospectively to summarize the clinical experience and evaluate the safety and effectiveness of thoracoscopic surgery.

## Materials and methods

### Patients

Clinical data for 92 patients with type III esophageal atresia in our hospital from January 2015 to December 2018 were analyzed retrospectively. The patients were grouped according to different operation methods, with 49 in group A undergoing thoracoscopic surgery and 43 in group B undergoing conventional surgery. All 92 patients had obvious clinical symptoms, combined with the results of three-dimensional reconstruction of the esophagus and chest CT, and all patients were diagnosed with type III esophageal atresia. All the patients’ preoperative clinical data are shown in Table 1; there were no statistically significant differences between the two groups. Patients met the inclusion criteria if they presented with type III esophageal atresia. Patients were excluded from this study if they 1) had other types of esophageal atresia, 2) had a poor overall state of severe hepatic and renal insufficiency, or 3) refused to sign the consent form for surgery or refused to comply with the follow-up schedule.

**Table 1:** General data comparison of two groups

	Group A	Group B	*P* value
Gestational age (weeks)	37.8 ± 3.4	38.6 ± 4.5	0.846
Age (days)	1.8 ± 0.6	1.6 ± 0.7	0.954
Body weight (g)	2887 ± 557	2665 ± 612	0.913
Boys/Girls	28/21	24/19	0.802
Premature infants	14	12	0.944
Congenital anal atresia	2	1	0.649
Uronephrosis	5	2	0.316

We have undergone thoracoscopic surgery since 2013, and by 2015 we have operated on 30 cases and have passed the learning curve. A total of one chief physician, one deputy chief physician and three attending physicians were involved in these operations.

Severe pneumonia was diagnosed, upon the symptoms of irritating or lethargy, refusing to eat, depression of lower chest wall and cyanosis. Pneumothorax was diagnosed upon the result of chest X-ray. Anastomotic leakage was diagnosed upon symptoms, such as dyspnea, pale complexion and fever, and when viscous mouth water, milk, or contrast medium (esophagogram) were found in the chest drainage tube. Anastomotic stenosis was diagnosed upon symptoms of dysphagia and the result of esophagogram.The healing time of the upper abdominal incision was generally 7–9 days. If the incision was still not healed after 9 days, we consider that to be poor wound healing. Chest wall deformity was diagnosed upon symptomsof locally obviously dented chest. The cost was included the all costs from admission to discharge.

### Thoracoscopic surgery

Body position: The left slope position and the right hand raised to the side of the ear.
Three incision: One 0.5 cm incision in line with the right subscapular angle flattened to the fourth intercostal. Two 0.3 cm incision on the third and sixth intercostal lines of the right axillary and posterior axillary lines.
The pleural adhesion was separated, and the tracheoesophageal fistula was found in the posterior wall of the bifurcation of the left and right trachea.
4.Ligating and cutting off the tracheoesophageal fistula.
Suturing at both ends of esophagus.
Checking bleeding and air leakage.
Closing the incision and inserting the thoracic drainage tube.

### Traditional surgery

The type III esophageal atresia operation was completed via an incision on the fifth intercostal posterolateral and anterior to the axillary front and back of the subscapular angle, respectively.

### Postoperative care

After operation, lifting the jaw to reduce the tension of esophageal anastomosis. Ventilator assisted breathing. After the tspontaneous breathing recover, stoping ventilator. According to the severity of pneumonia and bacterial culture selected antibiotics,and the anti-infection treatment was done until the pneumonia controled.

Start eating in the gastric tube 2–3 days after operation. Removing the drainage tube after no obvious drainage fluid.

### Statistical analysis

Continuous data are presented as the mean ± standard deviation and range. Clinical parameters between the two groups were compared with the independent samples t-test. The χ2 or Fisher’s test was used to categorize variables. A *p* value of < 0.05 was defined a statistically significant.

## Result

All operations were successful, with no conversion to thoracotomy for patients undergoing thoracoscopic surgery. There was no significant difference in preoperative data, operation time, postoperative extubation stomach tube time or hospital expenses between the two groups (*P* > 0.05). However, group A was superior to group B in terms of mechanical ventilation time, intensive care time, postoperative hospitalization time, thoracic drainage volume, blood transfusion volume and surgical incision length, and the difference was statistically significant (*P* < 0.05). (Table 2).

**Table 2:** Comparison of intraoperative and postoperative date between the two groups

	Group A	Group B	*P* value
Operation time (min)	153.8 ± 27.3	181.2 ± 30.4	0.712
Mechanical ventilation time(h)	55.7 ± 11.4	75.6 ± 19.2	0.042
intensive care time(d)	3.6 ± 1.8	4.7 ± 2.0	0.045
thoracic drainage volume (ml)	62.7 ± 25.5	125.4 ± 46.1	0.020
blood transfusion volume (ml)	30.5 ± 10.4	55.3 ± 22.7	0.043
Extubation stomach tube time (d)	10.5 ± 2.8	12.6 ± 3.5	0.834
surgical incision length (cm)	2.0 ± 0.5	8.0 ± 1.8	0.008
postoperative hospitalization time(d)	13.1 ± 2.2	16.8 ± 4.3	0.037
Hospital expenses(10,000 CNY)	3.7 ± 1.3	3.5 ± 1.1	0.912

**Fig. 1:**
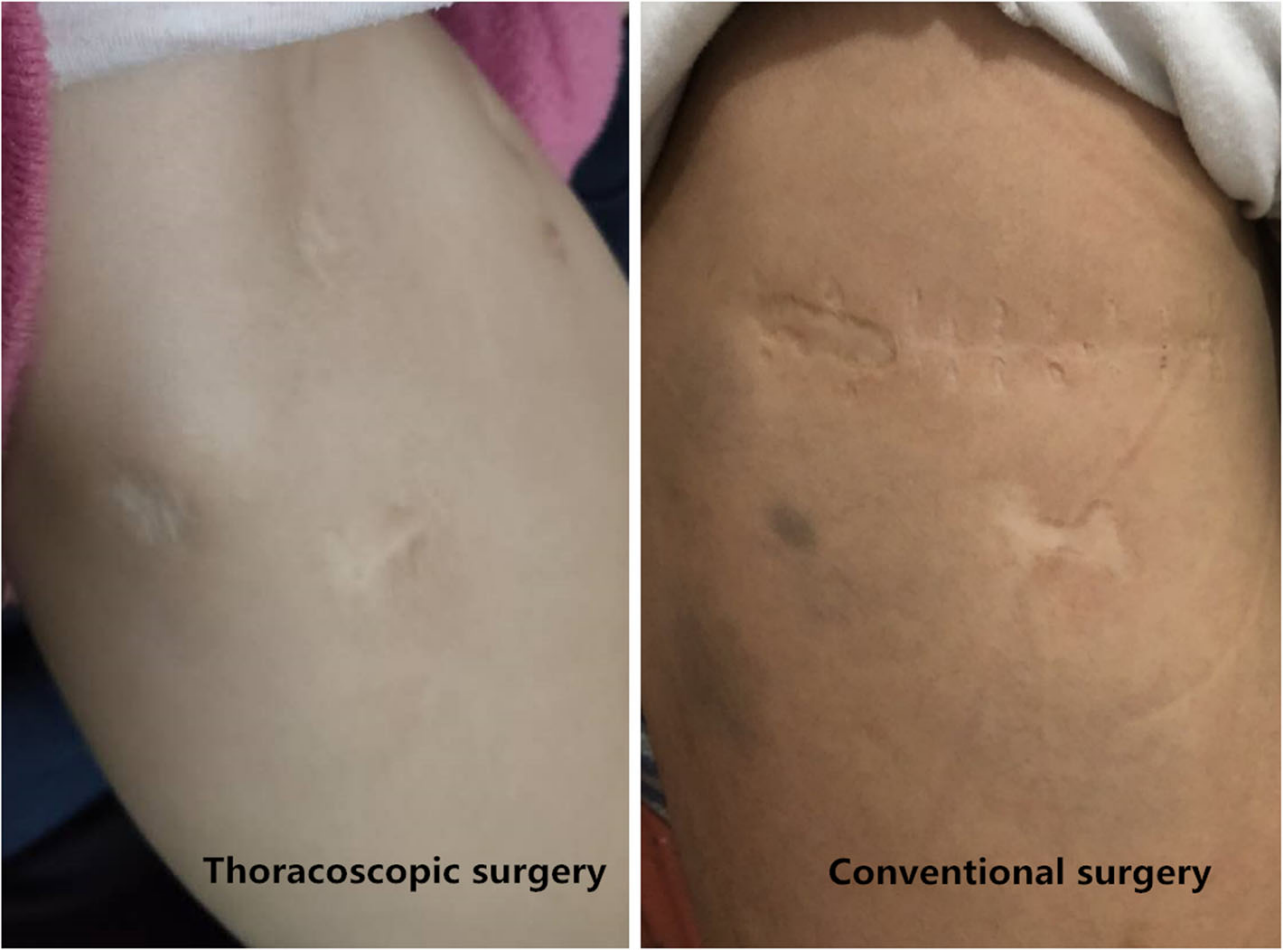
Incision of thoracoscopic surgery and conventional surgery

Among the postoperative complications, the incidences of postoperative severe pneumonia (8.2% vs 23.3%, *p* = 0.044), poor wound healing (2.0% vs 14.0%, *p* = 0.032) and chest wall deformity (0% vs 11.6%, *p* = 0.014) in group A were significantly lower than those in group B (Fig. 1). There was no significant difference in postoperative mortality (2.0% vs 2.3%, *p* = 0.926), pneumothorax (8.2% vs 7.0%, *p* = 0.830), anastomotic leakage (16.3% vs 14.0%, *p* = 0.752), anastomotic stenosis (20.4% vs 25.6%, *p* = 0.555) and gastroesophageal reflux (4.1% vs 2.3%, *p* = 0.636) between the two groups. There was no complications of tracheomalacia, signs of achalasia, oesophageal diverticulum in the two groups.

## Discussion

Traditional surgery with thoracotomy is a classical method for the treatment of congenital esophageal atresia with the advantage of more direct surgical vision and a larger operation space, which can more conveniently deal with accidents that occur during the operation. However, the traditional surgery has many disadvantages, such as a long incision and considerable injury to the chest wall, and chest wall deformity of the high shoulder blade, chest wall asymmetry, rib fusion, scoliosis and chest muscle dysplasia can easily occur. In contrast, thoracoscopic surgery only requires three 5-mm holes on the chest wall to complete the operation, and the incision is very small; thus, the injury to the chest wall, intercostal muscles and nerves is minimal, which can significantly reduce the incidence of chest wall deformity and surgical scarring. The data from our study showed that the incidence of chest wall deformity and the length of incision in thoracoscopic surgery were significantly lower than in those who underwent the traditional open surgery (*P* < 0.05). The results was similar to the findings of Suzuki M [14], Bastard F [15] and Lawal TA [16]. Their findings also suggest that thoracoscopic surgery has a smaller scarring and smaller incidence of chest wall deformities.

Thoracoscopy can provide an enlarged and clear visual field, which is beneficial to the identification and separation of tissues and organs and can help the operator perform fine anatomical operations and identify fine bleeding points in the operation field and accurately stop bleeding in a timely manner. Therefore, bleeding of the surgical wound is obviously reduced, as is that of the wound after the operation, and the blood products to be infused during and after the operation can also be reduced. In this study, the thoracic drainage volume and transfusion volume were 62.7 ± 25.5 ml and 30.5 ± 10.4 ml in group A, respectively, which were significantly lower than those in group B (125.4 ± 46.1 ml and 55.3 ± 22.7 ml), with a significant difference between the groups (*P* < 0.05). Thoracoscopic surgery reduces the risk of blood transfusion and the cost of treatment. Moreover, the distal esophagus can be clearly distinguished by moving the thoracoscopic lens, which can dissociate a longer esophageal, up to 2 cm above the thoracic entrance and downward to the esophageal foramen. The tension of the anastomotic orifice was reduced, and the difficulty of anastomotic anastomosis and the incidence of anastomotic leakage were also reduced [17]. At the same time, assistants, anesthesiologists and nurses have the same view as the surgeon, which can be conducive to surgical cooperation and communication.

Most children with type III congenital esophageal atresia have aspiration pneumonia before surgery. In addition to the impact of surgical trauma, surgical pain, intraoperative and postoperative bleeding, and blood transfusion, postoperative pulmonary infection is easily aggravated. Pulmonary infection is an important factor affecting the prognosis of children with esophageal atresia [18, 19]. Thoracoscopic surgery has the advantages of reduced trauma, bleeding and blood transfusion, and it can better maintain the integrity of the chest. Moreover, it can greatly reduce postoperative pain, help children cough and recover lung function after the operation, and speed the recovery of overall body function after the operation, which allows children to come off the ventilator early and promotes the absorption of pulmonary inflammation. In our study, postoperative severe pneumonia, mechanical ventilation time, intensive care time and postoperative hospitalization time in the thoracoscopic surgery group were better than those in the traditional operation group.

Although the operation for congenital esophageal atresia using a thoracoscope has many advantages, there are some disadvantages to this operation. The feel of the space with thoracoscopic surgery is different from that of the traditional surgery, and the operator must overcome the inertia of the traditional surgery operation to facilitate the operation. In newborns, the volume of the chest cavity is small, and the visual field and operation space of thoracoscopy are also small, such that the “chopstick effect” of the eyepiece and separation forceps easily appears in the narrow space [20]. Therefore, the difficulty of congenital esophageal atresia surgery via thoracoscopy is large, and the learning curve is steep. The studies showed that the study curve of esophageal atresia was 20–40 cases [21, 22]. However, the safety and effectiveness of the thoracoscopic operation gradually improve with the development of the thoracoscopic technique and improvement in the technique of the surgeon. Thoracoscopic surgery has become an effective treatment method for esophageal atresia combined with tracheoesophageal carcinoma [23]. Our center began endoscopic surgery early. At present, there have been more than 100 cases of thoracoscopic and laparoscopic surgeries, which have contributed to the skillful completion of all types of thoracoscopic surgery in children. In our study, the operating time for the thoracoscopic group was similar to that for the conventional operation group (*P* > 0.05). In general, a detailed assessment of the condition of the child should be performed before the operation to be fully prepared. Furthermore, a minimal operation should be carried out, and good communication with the anesthesiologist should be ensured to provide a high-quality thoracoscopic operation.

There are several limitations to this study. First, this was a retrospective study with a small sample size. Second, this was a single-center study, and more research from multiple centers is needed to assess the effectiveness and complications of this technique. Third, the follow-up period of this study was short, and a longer-term follow-up period is needed.

## Conclusion

The operation of type III esophageal atresia using a thoracoscope has the same safety and clinical effectiveness as traditional surgery and has the advantages of smaller incision and chest wall deformity.

## Data Availability

The datasets used and analysed during the current study are available from the corresponding author on reasonable request.
